# A Review on the Delivery of Plant-Based Antidiabetic Agents Using Nanocarriers: Current Status and Their Role in Combatting Hyperglycaemia

**DOI:** 10.3390/polym14152991

**Published:** 2022-07-24

**Authors:** Husna Zolkepli, Riyanto Teguh Widodo, Syed Mahmood, Norazlinaliza Salim, Khalijah Awang, Noraini Ahmad, Rozana Othman

**Affiliations:** 1 Department of Chemistry, Faculty of Science, Universiti Malaya, Kuala Lumpur 50603, Malaysia; ansuhzal117@gmail.com (H.Z.); khalijah@um.edu.my (K.A.); 2 Department of Pharmaceutical Technology, Faculty of Pharmacy, Universiti Malaya, Kuala Lumpur 50603, Malaysia; riyanto@um.edu.my (R.T.W.); syedmahmood@um.edu.my (S.M.); 3 Department of Chemistry, Faculty of Science, Universiti Putra Malaysia, Serdang 43400, Malaysia; azlinalizas@upm.edu.my; 4 Integrated Chemical Biophysics Research, Faculty of Science, Universiti Putra Malaysia, Serdang 43400, Malaysia; 5 Centre for Natural Products Research and Drug Discovery (CENAR), Universiti Malaya, Kuala Lumpur 50603, Malaysia; 6 Department of Pharmaceutical Chemistry, Faculty of Pharmacy, Universiti Malaya, Kuala Lumpur 50603, Malaysia

**Keywords:** antidiabetic, plant extract, nanocarriers, lipid nanoparticles, metallic nanoparticles

## Abstract

Diabetes mellitus is a prevalent metabolic syndrome that is associated with high blood glucose levels. The number of diabetic patients is increasing every year and the total number of cases is expected to reach more than 600 million worldwide by 2045. Modern antidiabetic drugs alleviate hyperglycaemia and complications that are caused by high blood glucose levels. However, due to the side effects of these drugs, plant extracts and bioactive compounds with antidiabetic properties have been gaining attention as alternative treatments for diabetes. Natural products are biocompatible, cheaper and expected to cause fewer side effects than the current antidiabetic drugs. In this review, various nanocarrier systems are discussed, such as liposomes, niosomes, polymeric nanoparticles, nanoemulsions, solid lipid nanoparticles and metallic nanoparticles. These systems have been applied to overcome the limitations of the current drugs and simultaneously improve the efficacy of plant-based antidiabetic drugs. The main challenges in the formulation of plant-based nanocarriers are the loading capacity of the plant extracts and the stability of the carriers. A brief review of lipid nanocarriers and the amphipathic properties of phospholipids and liposomes that encapsulate hydrophilic, hydrophobic and amphiphilic drugs is also described. A special emphasis is placed on metallic nanoparticles, with their advantages and associated complications being reported to highlight their effectiveness for treating hyperglycaemia. The present review could be an interesting paper for researchers who are working in the field of using plant extract-loaded nanoparticles as antidiabetic therapies.

## 1. Introduction

Diabetes mellitus (DM) is a common metabolic disease and non-infectious endocrine disorder that is associated with hyperglycaemia or high blood glucose levels, which are caused by the body’s impaired ability to metabolise glucose [1,2]. The number of DM cases continues to rise worldwide and it is expected that about 693 million people will suffer from DM between 2017 and 2045 [3]. This situation is mainly due to the rise in the incidence of type 2 diabetes. Type 2 diabetes is one of the main types of DM, while the other is type 1 diabetes [4]. Type 1 diabetes is an autoimmune disease in which insulin deficiency usually occurs due to the destruction of pancreatic beta cells [5].

Type 2 diabetes is a widespread metabolic disorder that results from various factors, including defective insulin secretion, the insulin resistance of insulin-sensitive tissues, ageing and environmental factors, such as stress and obesity [6,7]. The usual symptoms of DM include polyuria, polydipsia, weight loss and blurred vision [4]. In addition, patients with chronic hyperglycaemia usually suffer from growth impairment and are vulnerable to some infections. In patients with higher levels of glycaemia (blood glucose level), DM affects various systems in the body after a long period. Long-term complications include blindness, neuropathy, exocrine gland insufficiency, kidney failure and foot amputation [8]. DM also poses a significant risk for hypertension and could cause cardiovascular diseases, such as heart failure.

**Figure 1:**
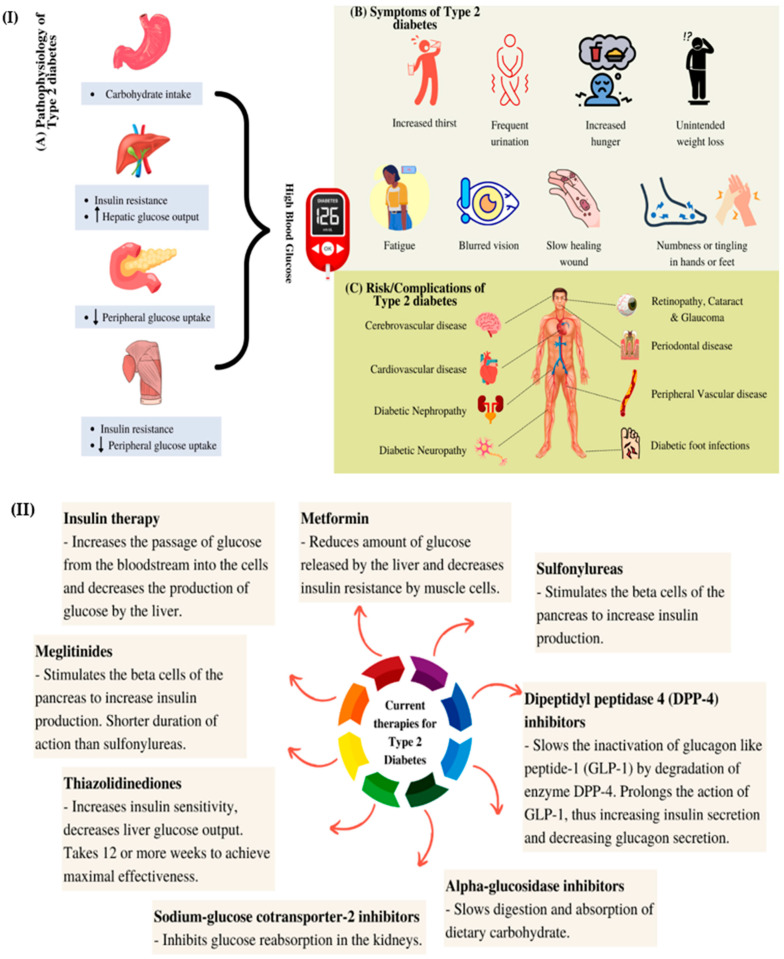
A schematic diagram representing (**I**) the aetiology and development of type 2 diabetes and (**II**) the current therapies for type 2 diabetes.

To treat patients with type 2 diabetes, both oral and injectable drugs are available [9]. Treatment should not be delayed and patients are advised to start pharmacotherapy to reduce the risks of irreversible microvascular complications, such as retinopathy [1]. A lifestyle change should accompany treatment with a single oral drug (monotherapy). There are currently ten classes of oral antidiabetic drugs that are available, including biguanides, sulfonylureas, meglitinide, thiazolidinedione and dipeptidyl peptidase 4 inhibitors [9]. A diagram of the aetiology, development and treatment of type 2 diabetes is illustrated in Figure 1 I,II.

The antidiabetic drugs in each class can be used as monotherapy or in combination with drugs from other classes. Metformin from the biguanide class is the most popular oral antidiabetic drug. It is always used as a first-line drug for diabetes due to its high efficacy, good safety profile, low price and lack of significant long-term side effects [9]. However, metformin does have some common side effects, such as diarrhoea and nausea. Even though it rarely occurs, patients who are prescribed with metformin may suffer lethal complications, such as lactic acidosis. Other antidiabetic drugs also have side effects, such as hypoglycaemia from sulfonylureas, weight gain from thiazolidinediones and acute pancreatitis from dipeptidyl peptidase 4 inhibitors [9]. All of the side effects and long-term treatments that are associated with the drugs have led to an increasing demand for efficacious, safe (few side effects) and affordable agents for the treatment of diabetes. Herbal medicines have been traditionally used to treat many diseases, including diabetes [10]. To ensure the efficacious drug delivery of phytochemicals that are present in plant extracts for the treatment of type 2 diabetes, various strategies have been employed, including nanocarrier-based therapy models, such as liposomes, niosomes, solid lipid nanoparticles and nanoemulsions. Hydrogels are also known for delivering bioactive agents to counter chronic conditions, such as diabetic wounds. They possess high water contents and can be developed using state-of-art designs that change according to temperature and pH [11,12].

This review reports and discusses plant extract-based nanocarriers for antidiabetic therapy.

## 2. Natural Active Agents in Nanocarriers

Since ancient times, many plant-based extracts and isolated bioactive compounds have been used across the world as therapeutic agents for the prevention and treatment of diseases and ailments [13]. Any plant-based products that are used to preserve or recover health are classified as herbal medicines [14]. About 200 years ago, herbal medicines dominated most medicinal practices. The use of herbal medicines started to decline in the 1960s, especially in the Western world, due to the introduction of allopathic medicines [15]. However, herbal medicines have been regaining public interest and have been slowly becoming more popular for various reasons, including the claims regarding the effectiveness of herbal medicines, changes in consumer preferences for natural medicines, the high costs and adverse effects of modern medicines and improvements in herbal medicines with the development of science and technology [16]. Research into identifying the chemical compounds from medicinal plants and their common uses may lead to new innovative drugs with fewer adverse effects than existing drugs [17]. It has been reported that medicinal plants, such as bitter melon (*Momordica charantia*), ivy gourd (*Coccinia grandis*) and ginseng, can be used to treat diabetes and more than 400 plant species with hypoglycaemic activity have been identified [18,19]; a few of these plants are listed in Table 1. The development of new antidiabetic drugs from plants has been attracting more attention as plants contain bioactive constituents that could have positive effects on the treatment of diabetes mellitus.

**Figure 2:**
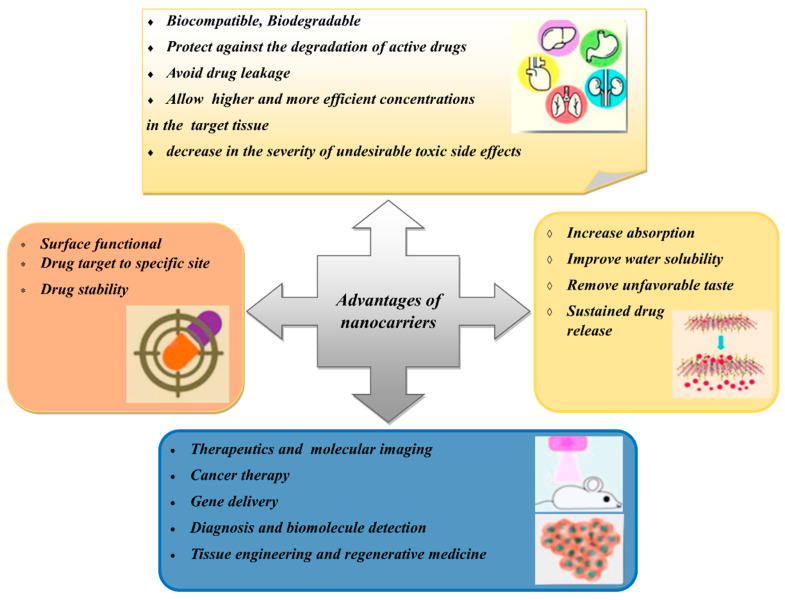
Advantages of using nanocarriers for plant extracts and bioactive constituents.

However, the application of herbal bioactive constituents and extracts in phytopharmaceuticals is still limited due to certain factors, such as unfavourable taste (e.g., bitterness), low solubility, poor permeability, physiological instability and low bioavailability [27,28]. To overcome these limitations, nanotechnological approaches have been explored as drug delivery mechanisms. Nanostructured drug delivery systems exhibit better physicochemical and biological properties than microscale drug delivery systems. The former systems have better optical properties, higher surface areas, better conductivity and improved interactions with biological molecules (Figure 2) [29]. Most of the bioactive compounds in plant extracts, such as flavonoids, tannins and terpenoids, are highly water-soluble. Thus, these compounds exhibit poor absorption as they cannot move across the lipid membrane [17]. This results in decreased bioavailability and efficacy [30]. By loading herbal medicines into nanocarriers, the absorption of the compounds can be improved, thus allowing for cellular uptake across the gastrointestinal wall of the bioactive compounds via passive transport [27].

Meanwhile, some natural compounds, such as caffeic acid and thymol, have low bioavailability due to their limited dissolution rates, which are affected by their low water solubility [31,32]. Encapsulating these compounds into nanocarriers can increase their surface areas, thereby improving their water solubility. Nanocarriers also allow for the controlled and sustained release of the natural compounds at the target site, which reduces the clearance, improves the therapeutic efficacy and reduces the adverse effects of the bioactive compounds [33]. Furthermore, natural compounds are better preserved when encapsulation occurs without any chemical reactions and nanocarriers can protect the compounds from gastric degradation [27,33]. All of the advantages of the use of nanocarriers for the delivery of drugs and herbal medicines are illustrated in Figure 2.

## 3. Types of Nanocarriers for Plant-Based Antidiabetic Extracts/Active Agents

**Figure 3:**
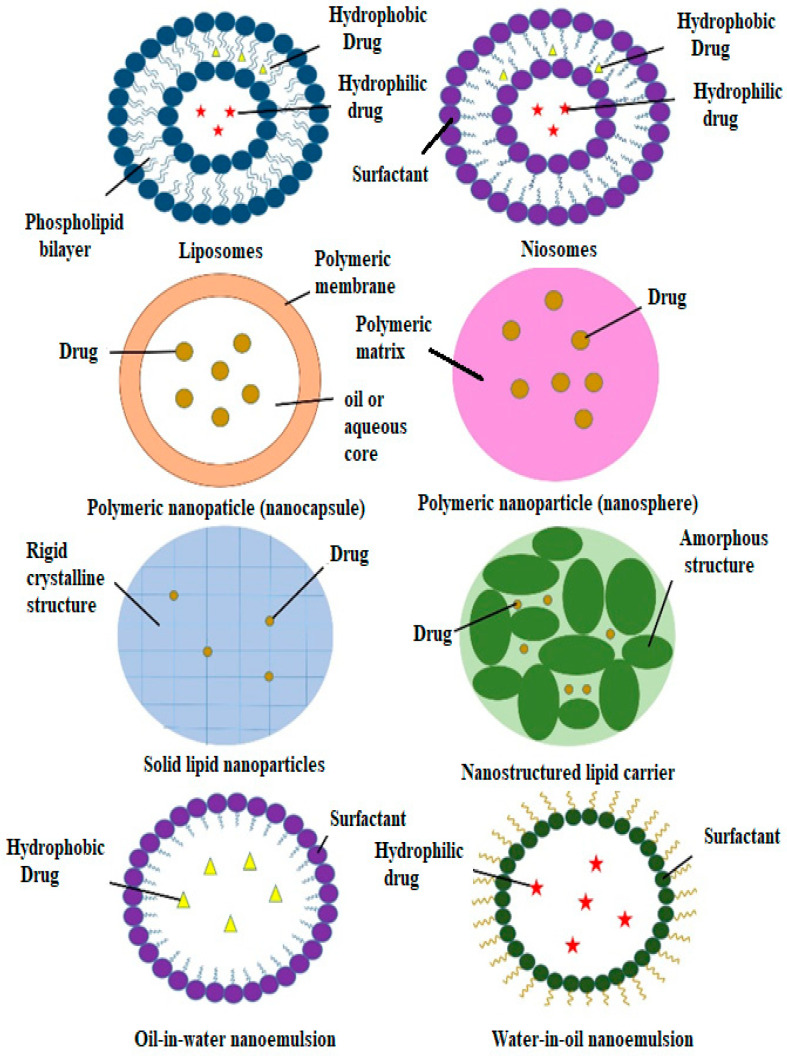
Different types of nanocarriers for antidiabetic agents.

Nanocarriers have sizes of between 1 and 100 nm and have been used as transporters to deliver active agents to target sites [34]. Nanocarriers are becoming more popular than conventional drug delivery systems due to their effectiveness, stability, improved drug bioavailability, target specificity and ability for the sustained release of the drug [35]. Furthermore, nanocarriers can carry various drugs with various biological properties. Many types of nanocarriers are available that can encapsulate natural compounds, as shown in Figure 3. However, this review only focuses on certain types of nanocarriers that have been studied for use with plant-based antidiabetic agents.

### 3.1. Liposomes

In 1965, phospholipid molecules were found to self-assemble by forming closed bilayer vesicles in water and were later termed liposomes [36,37]. Soon after that, liposomes were extensively studied for their potential as drug carriers through various administrative routes, such as parenteral, oral, pulmonary, nasal and transdermal routes [38]. Liposomes have an aqueous compartment that is enclosed by one or more cell-like lipid bilayers, which makes them suitable for cellular investigations. They also have essential cellular functions, such as motility and shape changes and the ability to impersonate the biophysical properties of living cells (Figure 2) [39,40,41]. Phospholipids are amphipathic molecules with water-loving (hydrophilic) and fat-loving (hydrophobic) parts [42]. The hydrophobic parts, which are the tails of the phospholipids, are repulsed by water molecules, which results in the self-assembly of liposomes through hydrophilic interactions, van der Waals interactions and hydrogen bonding interactions [28,43].

Due to the amphipathic properties of phospholipids, liposomes can encapsulate hydrophilic, hydrophobic and amphiphilic drugs [39,44]. Hydrophilic drugs are encapsulated in the aqueous part and hydrophobic drugs are encapsulated in the bilayer membrane between the tails of the phospholipids. In contrast, amphiphilic drugs are partitioned at the surface of the bilayers. The different positions of the drugs are due to their affinity to the different parts of the liposomes [44]. As well as the ability to control and sustain the release of drugs at specific sites, the liposomal membrane can also protect the encapsulated drugs from light, moisture and oxygen [45]. Liposomes can improve the physicochemical properties and onset time of the incorporated compounds and decrease their toxicity [46,47]. Liposomes fulfil the requirements of suitable drug carriers as they are biodegradable, biocompatible and stable in colloidal solutions [37].

When determining the final liposome structures, several crucial factors need to be considered: type and amount of phospholipid; the charge properties of the aqueous solution; hydration time; and the use of mechanical procedures and organic solvents [48]. As oral drug delivery systems, liposomes face some challenges that limit their potential as drug vehicles. Liposomes are vulnerable to the combined effects of the gastric acid, bile salts and pancreatic lipases within the gastrointestinal system [49]. Liposomes also have poor permeability to pass through the intestinal epithelia due to the relatively large size of their particles and the various epithelial barriers. Furthermore, it is difficult to mass-produce liposomes because of inconsistencies between batches [38]. The most important limitation of the use of liposomes as nanocarriers is their inability to retain active agents for prolonged periods compared to polymeric system nanocarriers [50].

The modulation of lipid compositions, surface coating, the addition of absorption enhancers and interior thickening have been carried out to develop better liposomes with special biological effects that could improve their application [38,51,52,53,54,55]. In addition, new apparatus and methods have been developed to overcome the mass production limitations, such as the continuous high-pressure extrusion apparatus and the high-speed dispersion method [56,57]. All new apparatus and methods need to be suitable for all routes of administration. The interactions between liposomes and cells depend on the composition, surface properties and type of liposomes and those of the interacting cells. Regarding these factors, liposomes are either endocytosed by the cells, adsorbed onto the cell surface or fused with the cell membrane. Some research has been conducted to study the potential of the use of liposomes as nanocarriers of plant-based antidiabetic agents (Table 2).

A recent study was carried out by Amjadi et al. (2019) to encapsulate betanin in a nanoliposome. Betanin is a bioactive compound that can be found in many plants, such as red beetroot (*Beta vulgaris*), amaranth (Amaranthaceae) and pitahayas (*Hylocereus undatus*) [58]. Betanin exhibits anti-inflammatory, anticarcinogenic and antidiabetic properties [59,60,61]. However, betanin shows less than 1% bioavailability through oral administration due to its insufficient oral absorption and fast degradation from its high reactivity under various conditions, such as temperature, pH and oxygen [62,63,64]. Betanin-incorporated lecithin nanoliposomes are prepared using the thin film hydration method to improve the therapeutic potency of the betanin [65]. These betanin-incorporated nanoliposomes have shown an excellent sustained release property in simulated gastrointestinal fluids (pH 1.2–7.4). This nanoformulation of betanin has exhibited a superior ability to free betanin in the treatment of hyperglycaemia, hyperlipidaemia and oxidative stress in streptozotocin-induced diabetic rats. This formulation has also shown therapeutic potential to reduce hyperglycaemia-induced tissue damage in the kidney, liver and pancreas. In a study that was performed by Bulboacă et al. (2019) [66], the same thin film hydration method was applied for liposome formulation using 1,2-dipalmitoyl-sn-glycero-3-phosphocholine (DPPC) and PEG-2000-DSPE. Curcumin-loaded liposomes have shown better results for lowering plasma glucose concentrations compared to free curcumin.

Furthermore, liposomal curcumin has also shown potential as a part of the treatment for reducing the risks of vascular diseases that are caused by diabetes by reducing the level of metalloproteinases. Metalloproteinase expression is always related to cell apoptosis in many pathologies. Hyperglycaemia is believed to induce the production of metalloproteinases, which can increase the risks of vascular complications [67,68].

Combined herbal extracts can also be used within a single nanoliposomal formulation to improve its therapeutic efficacy. For example, Gauttama and Kalia (2013) combined three extracts (bitter gourd (*Momordica charantia*) fruit extract, ashwagandha (*Withania somnifera*) root extract and fenugreek (*Trigonella foenum-graecum* seed extract) to form a polyherbal antidiabetic that was incorporated into liposomes [69]. These three plants have been scientifically proven to have antidiabetic properties. The vesicle system was developed using phosphatidylcholine and cholesterol. The polyherbal-encapsulated liposomes had improved antidiabetic efficiency compared to the free polyherbal formulation in streptozotocin-induced diabetic rats. The impact of the polyherbal-encapsulated liposomes on lowering blood glucose levels was similar to that of metformin.

**Table 2:** Nanoformulations for plant-based antidiabetic agents.

Type of Nanocarrier	Formulation (Ratio)	Active Compound	Model	Size Range (nm)	Remark	Ref.
Liposomes	Lecithin	Betanin	Streptozotocin-induced diabetic rats	40.06 ± 6.21	Increased hypoglycaemic activity; antihyperlipidemic activity; decreased oxidative stress	[65]
	DPPC, PEG-2000-DSPE and cholesterol (9.5:0.5:1)	Curcumin	Streptozotocin-induced diabetic rats	140	Increased hypoglycaemic activity; hepatoprotective effects; decreased oxidative stress	[66]
	Phosphatidylcholine and cholesterol (8:2)	*Momordica charantia, Trigonella foenum-graecum* and *Withania somnifera* extracts	Albino Wistar rats	1176 ± 5.6	Increased hypoglycaemic activity; antihyperlipidemic activity	[69]
Niosomes	Span 60 and cholesterol (1:1)	Lycopene	Alloxan-induced diabetic rats	202 ± 41	Increased hypoglycaemic activity; antihyperlipidemic activity	[70]
	Span 60, phospholipid 90G and cholesterol (9:4:1)	Embelin	Streptozotocin-induced diabetic rats	609–734	Increased hypoglycaemic activity; antioxidant efficacy	[71]
	Span 40 and cholesterol (1:2)	*Gymnema sylvestre* extract	Alloxan-induced diabetic rats	229.5 ± 30	Increased hypoglycaemic activity	[72]
Polymeric Nanoparticles	Poly-(ε-caprolactone) (PCL) and PLGA-PEG-COOH	Fisetin	In vitro assays	140–200	Better α-glucosidase inhibition than acarbose; scavenging capacity	[73]
	Eudragit RS100	*Phoenix dactylifera* seed oil	In vitro assays	207	α-amylase and α-glucosidase inhibition	[74]
	Chitosan	Curcumin	In vitro assays	74	Increased GLUT-4 levels	[75]
	Chitosan and alginate (3:1)	Naringenin	Streptozotocin-induced diabetic rats	150–300	Increased hypoglycaemic activity	[76]
	Chitosan and alginate (1:3)	Quercetin	Streptozotocin-induced diabetic rats	91.58	Increased hypoglycaemic activity	[77]
	Chitosan and gum arabic	Glycyrrhizin	Streptozotocin-induced diabetic rats	165.3	Increased hypoglycaemic activity	
	Chitosan and tripolyphosphate (4:1)	Ferulic acid	Streptozotocin-induced diabetic rats	51.2 ± 1.7	Increased hypoglycaemic activity; increased body weight	[78]
	Chitosan, gum Arabic and Tween 60	Glycyrrhizin	Streptozotocin- and nicotinamide-induced diabetic rats	181.4	Increased hypoglycaemic activity; reduced body weight and lipid levels	[79]
	Polyvinyl alcohol (PVA), Tween 80, gum-rosin polymer and oleic acid	Thymoquinone	Streptozotocin- and nicotinamide-induced diabetic rats	70.21	Increased hypoglycaemic activity; reduced body weight and lipid levels	[80]
	Gum rosin, PVA and lecithin	Thymoquinone	Streptozotocin-induced diabetic rats	36.83 ± 0.32	Increased hypoglycaemic activity	[81]
	PLGA	Quercetin	Streptozotocin-induced diabetic rats	179.9 ± 11.2	Increased hypoglycaemic activity; increased levels of catalase and superoxide dismutase	[82]
	PLGA	Pelargonidin	Streptozotocin-induced diabetic rats	91.47 ± 2.89	Increased hypoglycaemic activity; antihyperlipidemic activity	[83]
	PLGA, Pluronic F-127 and chitosan	Silybin	Streptozotocin-induced diabetic rats	184.6	Increased hypoglycaemic activity	[84]
	PLGA and PVA	Ethyl acetate	In vitro assays	365.7	α-amylase and α-glucosidase inhibition	[85]
	Tween 20 and propylene glycol	*Foeniculum vulgare* Mill. essential oil	Streptozotocin-induced diabetic rats	44–105	Increased hypoglycaemic activity	[86]
Nanoemulsions	Tween 20 and polyethylene (PEG) 400	*Ipomoea reptans* extract	-	15.5 ± 0.8	-	[87]
	Lecithin	Resveratrol	Streptozotocin + nicotinamide-induced diabetic rats	248	Increased hypoglycaemic activity; prevention of weight loss	[88]
Solid Lipid Nanoparticles	Compritol, Tween 80 and Span 20	Myricitrin	Streptozotocin + nicotinamide-induced diabetic rats	76.1	Increased hypoglycaemic activity; antioxidant and anti-apoptotic effects	[89]
	Glycerol tripalmitate and soybean phospholipid	Berberine	Male rats	76.8	Increased hypoglycaemic activity; prevention of weight gain	[90]
Nanostructured Lipid Carriers	Precirol and miglyol (5:2)	Baicalin	Streptozotocin-induced diabetic rats	92 ± 3.1	Increased hypoglycaemic activity	[91]

### 3.2. Niosomes

Niosomes, as with liposomes, are vesicles with a bilayer membrane that encloses an aqueous compartment (Figure 3) [92]. However, niosomes are prepared using non-ionic surfactants instead of phospholipids [93]. Niosomes were first developed for cosmetic purposes by a cosmetic company (L’Oreal) in 1975. Later, extensive research was conducted to further the applications of niosomes in other areas, including pharmaceuticals and food [93,94]. Niosomes have the same advantages as liposomes for use as drug delivery systems.

Niosomes can encapsulate both hydrophilic and lipophilic compounds due to their bilayer membranes and their enclosed aqueous cores [95]. As with liposomes, hydrophilic drugs are encapsulated in the aqueous centre and hydrophobic drugs are encapsulated between the tails of the bilayer. Niosomes have been developed as an alternative to liposomes for drug delivery systems [93]. The drug encapsulation efficiency of niosomes is better than that of liposomes because of their lower concentrations of cholesterol [92]. Niosomes are also less expensive for mass production and do not require special storage conditions, such as inert atmospheres, freezing temperatures (−20 °C) and darkness, which are essential for the manufacture of liposomes. The non-ionic surfactants that are used in the preparation of niosomes are much more stable than the lipids that are used for liposome production in terms of physical and chemical stability [96].

Moreover, phospholipids are even less stable as they more readily undergo oxidative degradation. Hence, liposome preparation is expensive and unique handling methods are essential [97]. On the other hand, niosomes can extend the circulation of the incorporated drugs due to their longer shelf life [98]. Liposomes have shorter shelf lives than niosomes due to their lipid components, which rapidly undergo rancidification [92,98]. Table 2 shows the niosome formulations with hyperglycaemic active ingredients.

For the past 30 years, niosomes have been applied as drug vehicles to reduce crucial biopharmaceutical problems, such as drug insolubility, adverse effects, target specificity, drug bioavailability and poor chemical stability [94,99]. The surfactants that are used in the preparation of niosomes are biodegradable, non-toxic and non-immunogenic and produce better stability, compatibility and reduced toxicity compared to anionic or cationic amphoteric surfactants [97,100]. In addition, some negatively charged molecules, such as dicetyl phosphate (DCP) and phosphatidic acid, and positively charged molecules, such as stearylamine (SA), can be added to the formulation of niosomes to improve their drug loading, increase their efficacy and improve their stability [101].

Sharma et al. (2017) prepared lycopene-loaded niosomes using Span 60, which is a commonly used non-ionic surfactant [70]. Lycopene is a carotenoid that is found in tomatoes (*Lycopersicum esculentum*). It is red and has many health benefits, including the ability to lower blood glucose levels [102]. However, lycopene also has the potential to be oxidised due to the presence of unsaturated bonds in its structure, which make it vulnerable to heat and light [103,104]. Compared to liposomes, niosomes have better stability and they are processed under less stressful conditions for lycopene, thereby lowering the possibility of the degradation of lycopene [70]. Both lycopene-loaded niosomes and glibenclamide were orally administered to alloxan-induced diabetic rats. Even though the entrapment efficiency of the lycopene niosomes was only around 60%, they exhibited a similar efficacy to glibenclamide (an antidiabetic drug) in terms of lowering blood glucose levels. In the same study, the lycopene niosomes also showed potential for the treatment of hyperlipidaemia as the levels of cholesterol, triglycerides, low-density lipoproteins (LDLs) and very low-density lipoproteins (VLDLs) were significantly decreased.

Embelin-encapsulated niosomes are another herbal antidiabetic nanoniosomal formulation that were studied by Alam et al. (2018) [71]. Embelin is a bioactive compound that is present in *Embelia ribes*, which is more commonly known as false black pepper. This compound is responsible for many of the pharmacological activities of the herb [105]. Niosomes were prepared using Span 60, phospholipid 90G and cholesterol and the thin film hydration technique. The blood glucose levels of streptozotocin-induced diabetic rats were lowered after treatment with the embelin-encapsulated niosomes. The hypoglycaemia efficacy of the niosomes was comparable to that of repaglinide. *Gymnema sylvestre* extract-encapsulated niosomes (which is another plant-based nanoformulation) also exhibited antidiabetic properties [72]. *Gymnema sylvestre* has traditionally been used to treat diabetes [106]. One of the bioactive constituents in this plant (gymnemic acid) is known for its poor aqueous solubility, gastric instability and high cholesterol-binding affinity [107]. These properties can lower the absorption of gymnemic acid, which can simultaneously decrease the therapeutic efficacy of this plant extract and its products. Hence, using niosomes could alleviate these properties. Based on the treatment of alloxan-induced diabetic rats, *Gymnema sylvestre* extract-encapsulated niosomes succeeded in decreasing blood glucose levels more than the free extract. The efficacy of these nanoformulations was comparable to that of glibenclamide. In addition, niosome applications have been studied extensively in anticancer therapies [108,109,110,111].

### 3.3. Polymeric Nanoparticles

Polymeric nanoparticles are solid colloidal particles that range from 1 nm to 1000 nm and are made up of biocompatible polymers [112]. Polymers are large molecules that are made by chemically linking one or more different types of small units, which are known as monomers, to form a linear or branched chain [113]. Monomers can have any structure, as long as they have at least two functional groups to form an interaction with another monomer. Polymeric nanoparticles can be prepared using block copolymers that have at least two polymer chains with different hydrophobicity that spontaneously assemble into a core–shell structure in an aqueous solution. The hydrophobic blocks create the core to reduce their exposure to the aqueous environment, while the hydrophilic blocks form the outer shell to stabilise the core [114]. As delivery systems, polymeric nanoparticles can transport various molecules, including drugs, proteins, plasmids, DNA and small interfering RNA [115,116]. Polymeric nanoparticles can be designed to deliver drugs at specific sites and then release them at special rates to achieve the optimum therapeutic drug concentration while minimising the exposure of the drug to undesired locations [112,117]. Polymeric nanoparticles are more stable than other nanocarriers, such as liposomes and micelles, in gastrointestinal fluid. The establishment of a strong chemical bond provides polymeric nanoparticles with more rigid and stable structures [117,118].

By manipulating the structure and composition of the polymer, delivery properties can be changed as desired. Early polymeric nanoparticles were developed using non-biodegradable polymers, such as polyacrylamide and polystyrene. However, these polymers have been shown to cause many side effects, such as chronic toxicity and bad immunological reactions. Since then, biodegradable polymeric nanoparticles have become more popular [119]. Biodegradable synthetic polymers are more appealing than natural polymers. Natural polymers can release drugs rapidly from nanoparticles due to their inability to resist degradation in the body. They can only last for a few hours, whereas synthetic polymers can prolong the drug release as they can resist degradation and remain in the body for days or even weeks [118]. Surfactants are essential in the preparation of polymeric nanoparticles. Surfactants can act as stabilising agents and add to the formulation to achieve a nanosystem that has a good structure and can stabilise dispersion during procedures. These stabilising agents are also beneficial for lowering the surface tension of nanoparticles and increasing their affinity with lipid structures [120]. There are two major types of polymeric nanoparticles, depending on their internal structure: nanospheres and nanocapsules (Figure 3) [121]. Polymeric nanospheres have solid/mass polymeric matrices, while polymeric nanocapsules have liquid/solid cores that are surrounded by solidified polymeric shells [122]. Due to this structure, polymeric nanocapsules have received more interest than nanospheres for drug delivery system applications. Drug loading is more efficient with nanocapsules because of their liquid/solid cores and their polymeric shells, which allow the encapsulated drugs to be better protected from degradation [123]. Hence, polymeric nanoparticles could be potential carriers for active hypoglycaemic ingredients (Table 2).

Chitosan is biodegradable and biocompatible polymer, which is safe for human consumption [124]. It is one of the most common natural polymers that are used to develop polymeric nanoparticles [125]. Many natural antidiabetic polymeric nanoparticles have been developed by using chitosan. Panwar et al. (2018) encapsulated ferulic acid to form chitosan–ferulic acid nanoparticles [79]. Ferulic acid is a polyphenolic compound that exhibits antidiabetic properties by regulating various biochemical and physiological pathways that are involved in hyperglycaemia. It was discovered that ferulic acid could stimulate insulin secretion in healthy pancreatic cells while inhibiting lipid peroxidation, which led to improvements in the symptoms of hyperglycaemia [126,127]. Ferulic acid-loaded chitosan nanoparticles were prepared using an ionotropic gelation process to improve the ferulic acid bioavailability as ferulic acid has low bioavailability and therapeutic efficacy. The encapsulated ferulic acid demonstrated an extended plasma retention period. The ferulic acid-loaded nanoparticles displayed a better hypoglycaemic effect in streptozotocin-induced diabetic rats than free ferulic acid while simultaneously causing increases in the body weight and blood lipid levels of the rats. Chitosan nanoparticles were also synthesised by Chauhan et al. (2018) to encapsulate curcumin, which is a bioactive compound that is known for its antidiabetic properties [75]. The nanoparticle formulations demonstrated improved GLUT-4 translocation at the cell surface compared to free curcumin. Better GLUT-4 translocation at the cell surface increases glucose uptake, hence the potential to lower blood glucose levels.

Natural polymers, such as chitosan and alginate, are cost-effective polymers, but their solubility in gastric fluid has limited their potential as an oral drug delivery system [128,129]. However, two natural polymers can be combined to develop nanoparticles that have improved properties, such as high drug entrapment, better resistance against pH and the improved sustained release of active agents. Furthermore, alginate and chitosan can be combined to synthesise nanoparticles that have better protection against degradation. Both Maity et al. (2017) and Mukhopadhyay et al. (2017) synthesised nanoparticles using chitosan and alginate. Maity et al. encapsulated naringenin, which is a polyphenolic compound that is commonly found in citrus fruits and has exhibited hypoglycaemic properties [130].

In contrast, Mukhopadhyay et al. encapsulated quercetin, which is the most abundant bioflavonoid and has demonstrated low toxicity with various health benefits, including antidiabetic effects [131]. Both the naringenin-loaded and quercetin-loaded nanoparticles demonstrated better antihyperglycaemic properties than free solutions [76,77]. Compared to chitosan nanoparticles, chitosan–alginate nanoparticles have demonstrated increased entrapment efficiencies by more than 90%. Chitosan–gum arabic nanoparticles that were used to encapsulate active agents with antidiabetic properties have also demonstrated higher entrapment efficiencies than chitosan nanoparticles while significantly decreasing the blood glucose levels of diabetic rats [77,79].

Polymeric nanoparticles can be synthesised using natural and synthetic polymers. Biodegradable synthetic polymers, such as poly(lactic-co-glycolic acid) (PLGA) and poly(caprolactone) (PLC), have attracted more attention than natural polymers. Natural polymers can provide relatively fast drug release from nanoparticles; however, they are susceptible to decomposition within a few hours. Meanwhile, synthetic polymers can prolong drug release because they have better resistance to degradation, so they can last for days or even weeks within the body [132]. PLGA nanoparticles have been developed to incorporate many active antidiabetic agents, such as quercetin, pelargonidin and ethyl acetate [80,81,82,83]. The in vivo testing of the antidiabetic activities of quercetin-loaded PLGA nanoparticles and pelargonidin-loaded PLGA nanoparticles showed that they were more effective at lowering blood glucose levels than free active agents. At the same time, the ethyl acetate-loaded PLGA showed potential as an antidiabetic medicine during tests that were performed based on α-amylase and α-glucosidase assays. Cationic modifications can improve the payload delivery of biopolymers, such as PLGA. For example, PLGA–chitosan nanoparticles have been synthesised to improve the delivery of silybin, which is an antidiabetic agent [84]. Silybin has demonstrated hypoglycaemic properties by inhibiting gluconeogenesis in the liver and lowering the activity of glucose-6 phosphatase [133].

### 3.4. Nanoemulsions

Nanoemulsions are thermodynamically stable dispersion systems that are made up of two immiscible liquids, such as water and oil, which are mixed with suitable surfactants and co-surfactants to form an interfacial film (Figure 3) [134]. The selected surfactants can be non-ionic or ionic surfactants [135]. Based on the composition of the oil and water portions, nanoemulsions can be classified into three types: oil in water (O/W), water in oil (W/O) and bi-continuous. For oil-in-water (O/W) nanoemulsions, oil droplets are dispersed in the aqueous phase. Meanwhile, for water-in-oil (W/O) nanoemulsions, water droplets are dispersed in the oil phase. As for bi-continuous nanoemulsions, microdomains of oil and water are inter-dispersed within the system [136]. As drug delivery systems, nanoemulsions have similar advantages to other nanocarriers; for example, nanoemulsions can deliver drugs to specific sites, protect drugs against degradation and increase their bioavailability [134,136]. Furthermore, nanoemulsions can dissolve a large number of lipophilic drugs. When administered orally, nanoemulsions can provide a pathway that improves the drug release rate by increasing the systemic absorption and systemic bioavailability of the drug [137,138].

A study that was conducted by Mostafa et al. (2015) demonstrated that nanoemulsions that were prepared using Tween 20 and propylene glycol as co-surfactants could encapsulate *Foeniculum vulgare* essential oil [86]. *Foeniculum vulgare* is a medicinal plant with antifungal, antibacterial, antioxidant and antidiabetic properties [139,140,141]. To assess antidiabetic efficacy on an animal model, the essential oil-encapsulated nanoemulsion was administered via topical administration instead of the usual oral route. This route of administration is easier, bypasses hepatic metabolism and is more convenient for patients [142]. The entrapment efficiency of this nanoemulsion formulation was about 60%. Furthermore, this essential oil-encapsulated nanoemulsion demonstrated a remarkable decrease in plasma glucose levels compared to free *Foeniculum vulgare* essential oil. Tween 20 has also been used to develop another self-nanoemulsifying drug delivery system (SNEDDS), but polyethylene glycol (PEG) was used as a co-surfactant instead [87].

In addition, this nanoemulsion formulation was used to encapsulate *Ipomoea reptans* extract. The leaf extract of *Ipomoea reptans* has been proven to reduce the blood glucose levels of mice [143]. However, *Ipomoea reptans* extract has low absorption within systemic circulation, which reduced the bioavailability of the extract. By encapsulating the extract into nanoemulsions, the bioavailability of the extract can be improved. During various physical endurance tests, such as centrifugation, heating–cooling cycles and freeze–thaw cycles, *Ipomoea reptans* extract nanoemulsions have exhibited good stability without phase separation. Unfortunately, the antidiabetic efficacy of *Ipomoea reptans* extract nanoemulsions has not yet been assessed, neither in vitro nor in vivo. Table 2 summarises the nanoemulsion formulations with antidiabetic potential.

### 3.5. Solid Lipid Nanoparticles and Nanostructured Lipid Carriers

Solid lipid nanoparticles (SLNs) consist of solid lipid matrices with single layers of phospholipids. Many solid lipids, such as triglycerides, fatty acids and steroids, can be used in combination with various surfactants, which can produce steric stabilisation in the formation of SLNs [144,145]. SLNs were developed in the 1990s and are used as a substitute to other nanocarrier drug delivery systems, such as emulsions, liposomes and polymeric micelles [146]. SLNs combine the benefits of polymeric nanoparticles, emulsions and liposomes while averting their disadvantages. Similar to liposomes and nanoemulsions, the lipids that are used in the preparation of SLNs are biocompatible and less toxic than specific polymeric nanoparticles [147,148]. The solid lipid matrices, which are identical to those in polymeric nanoparticles, protect the encapsulated active agents against chemical degradation in biological environments and provide high flexibility in terms of the release properties of the drugs. SLNs are capable of encapsulating both lipophilic and hydrophobic drugs [149]. The lipophilic property of the solid lipid matrices enables these nanoparticles to incorporate hydrophobic drugs. It is expected that hydrophilic drugs would be poorly encapsulated due to their low affinity to these lipid matrices. However, the double emulsion/solvent evaporation method can achieve satisfactory loading efficiencies for hydrophilic drugs [150,151]. Other advantages of SLNs include the targeted and controlled release of the encapsulated drug and the improved bioavailability of the drug [152]. Table 2 shows studies on SLNs that have been loaded with antidiabetic plant compounds.

Nanostructured lipid carriers (NLCs) were then developed to overcome the potential limitations of SLNs [153,154]. The structural difference between NLCs and SLNs is shown in Figure 3. The potential limitations of SLNs include low drug loading capacity, drug expulsion after polymeric transitions and the relatively high water volumes of the dispersions [155]. NLCs are synthesised using spatially different lipids that are composed of different fatty acids, which lead to bigger spaces between the fatty acid chains and imperfect crystallisation. These also improve the drug loading [155]. However, drug expulsion can occur due to the ongoing crystallisation, which can be prevented by adding special lipids, such as hydroxyl octacosanol and isopropyl myristate.

In a study that was conducted by Mohseni et al. (2019) to assess the potential of resveratrol-loaded SLNs for improving insulin resistance in induced diabetic rats, soybean lecithin was used to prepare the nanoparticles [88]. The resveratrol-loaded SLNs were administered to the rats orally and the nanoparticles demonstrated a better hypoglycaemic effect than free resveratrol. In another study that was conducted by Ahangarpour et al. (2018), myricitrin-loaded and berberine-loaded SLNs were synthesised [89,90]. The myricitrin-loaded SLNs were prepared using compritol and oleic acid as the liquid lipids. In addition, surfactants were added to the formulation to improve the hydrophilicity of the nanoparticles. The myricitrin-loaded SLNs demonstrated comparable antidiabetic effects to metformin. As well as lowering blood glucose levels, the myricitrin-loaded SLNs also improved complications from hyperglycaemia.

Considering the disadvantages of SLNs and the low hydrophilicity of baicalin, NLCs were used instead to encapsulate baicalin in the research of Shi et al. (2016) [91]. Baicalin is one of the flavonoid compounds that can be extracted from *Scutellaria radix* and has many pharmacological properties, such as anticancer, antibacterial and antidiabetic properties [156,157,158]. Precirol and miglyol were used to synthesise the baicalin-loaded NLCs. Precirol was used as the solid lipid to create the external shell of the nanoparticles. At the same time, miglyol was applied as the liquid lipid to improve the entrapment efficiency of the baicalin in the nanoparticles [159]. With an entrapment efficiency of more than 80%, the baicalin-loaded NLCs were significantly better at lowering blood sugar levels compared to free baicalin.

## 4. Metallic Nanoparticles

The use of metallic nanoparticles has shown remarkable progress in biomedical sciences and can counter antibacterial, antidiabetic and anticancer activities. A decade of research on using metallic nanoparticles to entrap plant extracts has gained the interest of many researchers as it has presented remarkable results. Metallic nanoparticles possess unique properties, such as large surface areas, specialty functional groups, effective quantum self-assembly and the ability to conjugate with the drug of interest, which make them favourable for biotechnology, targeted drug delivery and potential in vivo imaging. Additionally, metallic nanoparticles have shown many advantages, such as manufacturing simplicity, reproducibility, economy, stability, environmental friendliness and high entrapment efficiency, which make them favourable candidates for various applications. The synthesis and fabrication of metallic nanoparticles primarily use metal and metal oxides, such as gold, silver, copper and titanium–cerium–zinc oxide. In nanoformulations that involve plant extracts, they act as stabilising and reducing agents [160]. The most common metallic nanoparticles to be studied are gold nanoparticles (Au NPs) and silver nanoparticles (Ag NPs). Gold nanoparticles are commonly synthesised using the Turkevich method, whereas silver nanoparticles are synthesised using the bio-reduction process [161]. Zinc oxide nanoparticles (ZnO) are very common metallic particles that are used due to their large binding energy [162]. Recent studies that have been published on metallic nanoparticles and their applications in antidiabetic therapies are illustrated in Table 3.

## 5. Characterisation of Nanocarriers

The physicochemical characterisation of nanocarriers has been known to be related to their efficacy and applications, including their size and its polydispersity index, morphology, surface charge, zeta potential, entrapment efficiency, drug release evaluation, compatibility and stability [178,179,180]. Size and polydispersity are important parameters in nanocarriers as they determine the drug release characteristics and the biodistribution and bio-elimination of nanocarriers. There are various methods to determine the size and polydispersity of nanocarriers, such as dynamic light scattering (DLS) and microscopic techniques, including atomic force microscopy (AFM) and centrifugal liquid sedimentation (CLS). The selection of the method is dependent on the expected size and population of the nanocarriers [178,179].

The morphology or shape of nanoparticles may affect their biodistribution, targeting efficacy and degree of cytotoxicity. This characteristic can be determined using scanning electron microscopy (SEM) or transmission electron microscopy (TEM) [180,181]. For metallic nanoparticles, compositional analysis is performed using energy dispersive X-ray spectroscopy (EDX) with a scanning electron microscope (SEM-EDX) [181]. Surface charge and zeta potential affect the stability of dispersion nanocarriers. These two parameters govern the repulsion between the nanocarriers (electrophoretic mobility distribution) and stability is achieved when the value of the zeta potential is at or above ±30 mV [180]. Dynamic light scattering instruments, such as Brookhaven (NanoDLS^®^ series), Microtrac (Wave II^®^ series) and Malvern (Zetasizer^®^ series), are used to determine the particle size and surface charge of nanocarriers [182]. Entrapment efficiency refers to the amount of drug that is encapsulated by nanocarrier and needs to be determined as it influences the release kinetics of the drug.

### 5.1. Drug Release Study

Evaluating the drug release characteristics of nanocarriers is very important as the results can be used to optimise the formulation and to predict the therapeutic efficiency and side effects. The most popular method that is adopted for this purpose is the dialysis method [180].

### 5.2. Compatibility and Stability Study

Nanocarriers are composed of various different chemicals and should be compatible and inert. Therefore, compatibility studies are required, which are commonly conducted using differential scanning colourimetry or X-ray diffraction. Nanocarriers need to be stable until the drug has reached the target site in order not to affect the efficacy of the drug. Therefore, predicting the stability of nanocarriers physically, chemically and in physiological environments is very important [180].

## 6. Future Perspectives

Nanocarriers have been gaining more attention within medicinal fields over recent years. In the treatment of diabetes, which can require continuous and prolonged treatment, patient compliance is crucial to achieve the treatment targets. Nanocarriers have been found to increase patient compliance and therapeutic efficacy by providing various routes for administration, concealing unfavourable tastes, improving the controlled release of drugs, increasing the stability of active agents and improving target specificity. Hence, the study of nanocarriers as antidiabetic agents has increased over recent years. Due to the various side effects of modern antidiabetic drugs, plant-based active agents have also been gaining more attention. They could provide similar effects to the modern drugs but with fewer side effects. Based on Table 2, most studies on plant-based antidiabetic active agent nanocarriers have focused on polymeric nanoparticles. This could be due to their more affordable nature and biocompatible components for developing polymeric nanoparticles, such as chitosan and alginate. Additionally, the up-scaling and mass production of polymeric nanocarriers are much easier than for liposomes or niosomes. Polymeric nanocarriers also exhibit better stability than other nanocarriers.

Regarding the outcomes of the studies, selected antidiabetic active agents demonstrated hypoglycaemic abilities and the ability to reduce issues that were caused by high blood glucose levels. Betanin and curcumin were two of the active antidiabetic agents that demonstrated the ability to reduce oxidative stress that was caused by hyperglycaemia [62,66]. Oxidative stress plays a crucial part in the development of diabetic complications. Pancreatic tissue has a low anti-oxidative capacity, which causes it to be more vulnerable to oxidative stress and eventually leads to the damage of pancreatic beta cells [159]. With the destruction of the pancreatic beta cells, patients would slowly lose their ability to secrete more insulin. By developing nanocarriers that can be loaded with natural product-based active agents, the pharmacokinetic and therapeutic efficacy of those active agents could be improved. As research continues, nanocarrier technology could offer new extensive biomedical opportunities and challenges. Furthermore, many plant-based antidiabetic active agents have other biological properties, such as antioxidant and antihyperlipidemic properties. Thus, there is the potential to use these nanocarriers in combination with treatments for other diseases. However, the data from the existing studies are still inadequate to further the research to the next level, especially regarding the long-term therapeutic effectiveness, the possibility of developing side effects during prolonged treatment and the stability of the formulations. Hence, more research needs to be conducted on these issues to maximise the potential of plant-based antidiabetic nanoformulations. In the case of metallic nanoparticles, a concern has been raised regarding their toxic natures and smaller sizes, which can cross the blood–brain barrier. A new modified metallic nanoparticle of the optimum size (150–250 nm) with higher entrapment and a lower toxic profile could be used for diabetic therapy. Nevertheless, there is no denying that the application of nanocarriers to deliver plant-based antidiabetic agents has opened new opportunities for the treatment of diabetes.

## 7. Conclusions

Currently, more than 400 plants have been clinically proven to have antidiabetic properties. Those plants have been slowly gaining attention as alternatives to allopathic medicines. Many studies have been conducted on nanocarrier formulations for antidiabetic bioactive compounds and extracts from plants to overcome the limitations of plant-based products, such as low solubility, poor permeability and low bioavailability. The best properties for countering antidiabetic effects have been shown by metallic nanoparticles and liposomes due to their versatile nature and their vast areas of application. The efficacy of these plant-based nanoformulations against hyperglycaemia-related conditions has been proven through both in vitro and in vivo assessments. Hence, nanocarriers for herbal antidiabetic medicines have a lot of potential as alternative treatments for diabetes mellitus. However, further research needs to be carried out to discover which nanocarriers could offer high therapeutic efficacy for managing diabetes and hyperglycaemia complications.

## Figures and Tables

**Table 1:** Medicinal plants with antidiabetic effects.

Plant Part	Scientific Name	Common Name	Antidiabetic and Other Biological Activities	Ref.
Bark	*Pterocarpus marsupium*	Indian kino tree	Antidiabetic and hypoglycaemic	[20]
Fruit	*Momordica charantia*	Bitter melon	Antidiabetic and hypoglycaemic	[21]
Leaf	*Gymnema sylvestre*	Gurmar	Hypoglycaemic and hypolipidemic	[22]
	*Catharanthus roseus*	Nayantara	Hypoglycaemic	[23]
	*Azadirachta indica*	Neem	Hypoglycaemic	[23]
	*Ocimum sanctum*	Holy basil	Antidiabetic and hypoglycaemic	[20]
	*Aloe vera*	Aloe vera	Antidiabetic, antihypercholesterolemic and antioxidative	[24]
Rhizome	*Curcuma longa*	Turmeric	Antidiabetic, antioxidant and anticholinesterase	[25]
Seed	*Allium sativum*	Garlic	Hypoglycaemic	[23]
	*Trigonella foenum-graecum*	Fenugreek	Antidiabetic and hypoglycaemic	[20]
Stem	*Tinospora cordifolia*	Giloy	Hypoglycaemic	[26]

**Table 3:** Metallic nanoparticles for plant-based antidiabetic agents.

Type of Nanocarrier	Plant Extract/Compound	Approximate Size Range (nm)	*In Vitro*/*In Vivo* Model	Outcome	Ref.
Zinc Oxide (ZnO)	Red sandalwood (RSW)	20 nm	α-Amylase and α-glucosidase inhibition assays with murine pancreatic and small intestinal extracts	ZnO–RSW conjugate showed 61.93% inhibition compared to ZnO nanoparticles and RSW, which showed 21.48% and 5.90% inhibition, respectively	[163]
Silver Nanoparticles (Ag NPs)	Bedu *(Ficus palmate*)	30 nm	α-Amylase and α-glucosidase assays	Inhibition of α-amylase IC_50_ showed by Ag NPs for *F*. *palmate* leaves (27 μg/mL) and inhibition of α-glucosidase IC_50_ showed by Ag NPs for *F. palmate* leaves (32 μg/mL)	[164]
ZnO NPs	*Andrographis* *paniculata*	Spherical 96–115 nm and hexagonal shapes of 57 ± 0.3 nm	α-Amylase inhibitory activity	IC_50_ values of the ZnO NPs (121.42 μg/mL) were lower than those of the *A. paniculata* leaf extract ZnNO_3_ (149.65 and 178.84 μg/mL, respectively)	[165]
Copper Oxide Nanoparticles (CuO NPs)	*Bacopa monnieri*	22 nm	STZ-induced diabetic mice	Blood glucose levels were reduced by about 33.66 and 32.19% in groups of mice that were treated with CuO NPs and CuO NPs + insulin, respectively	[166]
Ag NPs	*Phyllanthus emblica*	30–65 nm	Alloxan-induced diabetic mice	Oral administration of Ag NPs reduced glucose levels from 280.83 ± 4.17 to 151.17 ± 3.54 mg/dL	[167]
Gold Nanoparticles (Au NPs)	*Leucosidea sericea* total extract (LSTE)	6, 24 and 21 nm	α-Amylase inhibitory activity	Fraction LSTE 4 (F-1) Au NPs demonstrated the highest IC_50_ value of 1.88 µg/mL	[168]
ZnO NPs	*Areca catechu*	29 nm	Glucose uptake assay with yeast cells (*Saccharomyces cerevisiae*)	ZnO NP-treated yeast cells showed a decrease in uptake, which was attributed to antidiabetic activity	[169]
Au NPs	*Hylocereus polyrhizus* (Red Pitaya) Red Dragon Pulp	25.31 nm	α-Amylase inhibitory activity	Significant inhibition (IC_50_) of 40.07 ± 0.65, 22.02 ± 0.15 and 11.34 ± 0.11 at 200, 100 and 50 µg/mL, respectively	[170]
Copper Oxide Nanoparticles (CuO NPs)	*Areca catechu*	200–800 nm	α-Amylase (pancreatic) inhibition assay and glucose uptake with yeast cells	α-Amylase: the inhibition by CuO NP samples showed an IC_50_ value of 17.3049 at 100 μg/mL; Yeast model: percentage of glucose uptake by the samples showed a value of 70.81 at 250 μg/mL	[171]
Au NPs	*Dittrichia viscosa*	20 and 50 nm	STZ-induced diabetic adult male Sprague–Dawley rats	Significantly lower levels of hepatic PEPCK enzyme activity when treated with Au NPs compared to the diabetic group (*p* < 0.02)	[172]
Silver Nanoparticles	*Justicia wynaadensis*	30 nm–50 nm	Enzymatic activity of α-amylase	The IC_50_ value of the synthesised Ag nanoparticles was 493.87 µg/mL	[173]
Reduced Graphene Oxide (RGO—ZnO)	*Ocimum basilicum*	31 nm	α-Amylase and α-glucosidase enzyme inhibition assays	Inhibition activity increased by 67.12% for ZnO NPs and 72.41% for RGO–ZnO NCs at 600 µg/mL	[174]
Au NPs	*Citrus aurantifolia*	17–24 nm	α-Glycosidase inhibition results	Au NPs produced IC_50_ values of 43.51 and 130.32 μM	[175]
Nickel Oxide Nanoparticles (NiO NPs)	*Areca catechu*	5.46 nm	α-Amylase and α-glucosidase enzyme inhibition assays	The results showed α-amylase enzymes with IC_50_ values of 268.13 µg/mL	[176]
Platinum Nanoparticles (Pt NPs)	*Polygonum salicifolium*	1–3 nm	In vitro α-amylase and α-glucosidase inhibition	α-Amylase inhibitory IC_50_ was found to be 72 μg/mL; α-glucosidase inhibitory activity IC_50_ was found to be 53 μg/mL	[177]

## Data Availability

Not applicable.
